# Leveraging Machine Learning for Screening Metal-Organic Frameworks with Selective CO_2_ Recognition for Early Thermal Runaway in Lithium-Ion Batteries

**DOI:** 10.3390/nano16040245

**Published:** 2026-02-13

**Authors:** Xian Wei, Xin Li, Xiong Wang, Xiaoyan Liu, Chen Zhu

**Affiliations:** 1 College of Electronic and Optical Engineering & College of Flexible Electronics (Future Technology), Nanjing University of Posts and Telecommunications, Nanjing 210023, China; 2 College of Integrated Circuit Science and Engineering, Nanjing University of Posts and Telecommunications, Nanjing 210023, China; 3 ISCTE Business School, BRU-IUL, University Institute of Lisbon, 1649-026 Lisbon, Portugal

**Keywords:** thermal runaway warning, CO_2_ selective recognition, metal-organic frameworks, machine learning, multicomponent adsorption

## Abstract

The escalation of thermal runaway in lithium-ion batteries presents severe safety hazards that necessitate advanced monitoring protocols to ensure early warning of potential failures. Carbon dioxide (CO_2_) is released during preliminary decomposition well before catastrophic failure occurs, thereby providing a strategic advantage for early-stage warning. Consequently, identifying materials with high-selective CO_2_ recognition is an essential prerequisite for developing reliable sensing platforms. This study integrates Grand Canonical Monte Carlo simulations with Random Forest (RF) models to systematically screen 1470 MOFs from the CoRE-MOF 2019 database. The screening process evaluates selective CO_2_ recognition under multicomponent competitive adsorption conditions involving CO_2_, C_2_H_4_, and O_2_. The performance evaluation is based on working capacity, selectivity, and the trade-off between working capacity and selectivity (*TSN*). The RF model achieves high predictive accuracy, with tested *R*^2^ exceeding 0.92 on the test samples. Shapley Additive Explanations (SHAP) interpretability analysis identifies *Q*^0^*_st_*(CO_2_), *Q*^0^*_st_*(C_2_H_4_), *WEPA*, *K_H_*(C_2_H_4_), and *ETR* as key performance drivers. The results indicate that CO_2_ selectivity is constrained by the binding strength of competing C_2_H_4_. Optimal materials tend to have hard Lewis acid centers and polar inorganic clusters to minimize non-specific π-interactions with interfering species. Top-performing MOFs require balanced structural features, concentrating in moderate surface areas (965–1975 m^2^/g), narrow pore windows (*PLD* ≈ 4–7 Å, *LCD* ≈ 5.5–9.6 Å), high void fractions above 0.6, and low densities below 1.3 g/cm^3^. AJOTEY emerges as the optimal candidate with a *TSN* of 6.43 mol/kg, combining substantial working capacity (4.57 mol/kg) with strong selectivity (25.52). These results will accelerate the discovery of sensing materials and provide a practical pathway for MOF-based CO_2_ sensor development to enhance lithium-ion battery safety.

## 1. Introduction

Lithium-ion batteries are central to the global shift towards electric mobility and stationary energy storage systems [1,2]. However, thermal runaway remains a critical safety concern that can trigger catastrophic failures including fires and explosions [3,4]. The thermal runaway process unfolds in several stages, each associated with distinct gas emissions [5]. Carbon dioxide (CO_2_) is the primary gas released during the early stages, emerging at temperatures as low as 80–120 °C when the solid electrolyte interphase starts to break down. This initial CO_2_ emission offers a critical opportunity for intervention before the temperature rises further [6]. In contrast, hydrogen (H_2_) is released in large quantities above 200 °C. This occurs in the later stages, when the electrolyte decomposes violently. Most existing safety monitoring strategies therefore rely on H_2_ detection, by which point the battery has already entered a highly dangerous state [7,8]. By the time H_2_ reaches detectable levels, thermal runaway has already progressed significantly, making intervention extremely difficult. Recent studies have shown that CO_2_-selective systems can provide warning signals several minutes earlier than H_2_-based approaches [9]. This early warning provides time for cooling, power disconnection, or evacuation [10]. Taken together, these results highlight CO_2_ recognition as a more effective route for early-stage thermal runaway warning compared with conventional H_2_-based strategies. This effectiveness places the primary emphasis on developing materials with intrinsic and selective CO_2_ recognition capability.

Metal-organic frameworks (MOFs) are porous crystalline materials with high specific surface areas and tunable pore structures, making them promising candidates for CO_2_ sensing in battery safety applications [11]. When employed as gas-responsive materials, their performance is governed by sensitivity, selectivity, response kinetics, and long-term stability. These properties can be systematically tuned through rational structural design. For example, HKUST-1 utilizes open Cu^2+^ sites to enable strong chemisorption [12,13], while NH_2_-Mg-MOF-74 employs ethylenediamine groups to amplify capacitive response [14]. Due to the complex gas mixtures released by batteries, selectivity is crucial for early thermal runaway detection, as CO_2_ must be distinguished from interfering species like C_2_H_4_ and O_2_. Some MOFs have demonstrated promising selectivity through molecular sieving effects. ZIF-8 with its narrow 3.4 Å pores selectively admits CO_2_ while excluding larger molecules. MUF-16 shows inverse selectivity over C_2_H_4_ with uptake ratios reaching 15.6 [7,10,15]. Despite these successes, identifying the most suitable MOF candidates from the vast structural landscape remains a formidable challenge.

Addressing this challenge increasingly relies on data-driven strategies that enable efficient exploration of large materials spaces. Currently, data-driven methodologies are revolutionizing battery science. Advanced deep learning techniques have been extensively applied to enhance battery thermal management and realize early safety warnings [16], while emerging large language models are accelerating material discovery and experimental design by mining vast databases [17]. Over 100,000 MOF structures have been synthesized to date, with millions more theoretically possible through different metal-ligand combinations [18,19,20,21,22]. Experimentally testing each candidate for CO_2_ selectivity against interfering gases like C_2_H_4_ and O_2_ would be prohibitively time-consuming and expensive [23,24,25]. Traditional computational screening relies on high-throughput molecular simulations such as Grand Canonical Monte Carlo (GCMC) to calculate gas adsorption properties [26]. While GCMC provides accurate predictions, comprehensive evaluation of large-scale databases like CoRE-MOF with over 14,000 structures remains computationally prohibitive [27,28,29]. Machine learning (ML) offers a transformative solution by learning structure-property relationships from limited simulation data [30]. Models trained on structural descriptors such as pore size, surface area, and functional group distribution can rapidly predict adsorption selectivity for thousands of MOF candidates [31,32]. This computational strategy enables efficient identification of MOFs optimized for detecting the early CO_2_ signature of thermal runaway in battery systems.

**Scheme 1:**
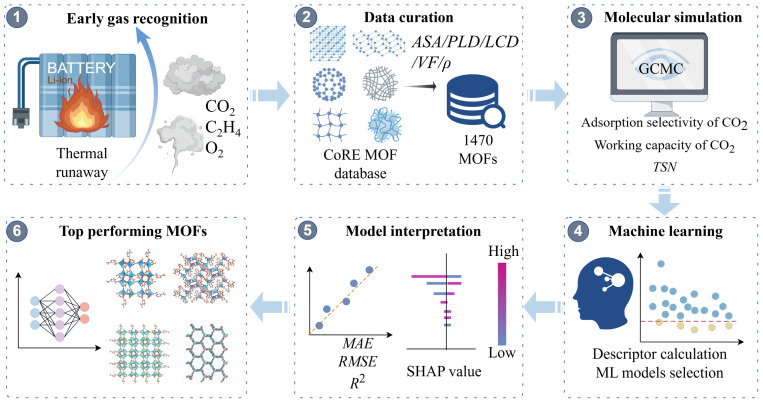
Computational workflow for ML-guided screening of MOFs for CO_2_ recognition in battery thermal runaway applications by figdraw.com.

In this study, GCMC simulations combined with Random Forest (RF) models were used to systematically screen 1470 MOF materials for CO_2_ recognition in battery thermal runaway scenarios (Scheme 1). We assess working capacity, selectivity, and overall performance under multicomponent competitive adsorption conditions, which simulate the complex gas mixtures released during early battery failure. Analysis of key structural descriptors reveals non-linear relationships between MOF architecture and CO_2_ adsorption performance. Model interpretability techniques identify the dominant physicochemical factors governing CO_2_ selectivity against interfering gases. High-performance MOF candidates for selective CO_2_ recognition were identified. Such capabilities are crucial in the early stages of lithium-ion battery thermal runaway, offering a new pathway for developing advanced battery safety systems.

## 2. Computational Details

### 2.1. Database and Screening Process for MOFs

Structural and chemical descriptors were calculated for 1470 MOFs as listed in Table S1. The MOF dataset was initially selected from the CORE-MOF 2019 database totaling 6603 MOFs. To characterize the geometric and size attributes of the internal pores in MOFs, five structural descriptors were computed like largest cavity diameter (*LCD*), pore limiting diameter (*PLD*), accessible surface area (*ASA*), density (*ρ*), and void fraction (*VF*). These descriptors were calculated using the Zeo++0.3 software, with He and N_2_ as probes (kinetic radius: 1.32 Å and 1.86 Å, respectively) to estimate *VF* and *ASA*.

In addition to these structural descriptors, adsorption enthalpy and Henry’s coefficients (*K_H_*) under infinite dilution conditions were incorporated as chemical descriptors of the MOFs. Furthermore, various types of atomic counts were extracted from the crystal structures of the MOFs, from which additional chemical descriptors were derived. These descriptors include the type and number of each atom, total unsaturation degree, metal percentage, oxygen-to-metal ratio, electronegativity ratio, weighted electronegativity per atom, and nitrogen-to-oxygen ratio.

The screening of the MOFs was based on the following criteria: (1) *PLD* > 3.3 Å, (2) 950 m^2^/g < *ASA* < 5000 m^2^/g, (3) 0.1 < *VF* < 0.7, and (4) *K_H_*(H_2_O) < 2.6·10^−6^ mol/(kg·Pa). This process resulted in the selection of 1470 MOFs for further analysis.

### 2.2. Simulation Details for GCMC

In the initial stage of thermal runaway in lithium-ion batteries, the temperature continuously rises due to the decomposition of the solid electrolyte interphase (SEI) between 80–120 °C, leading to the first reaction. When the temperature approaches 90 °C, the decomposition of SEI begins to release a significant amount of heat and gases (CO_2_, C_2_H_4_, and O_2_) [33]. The reaction is as follows:

(CH_2_OCO_2_Li)_2_ → Li_2_CO_3_ + C_2_H_4_↑ + CO_2_↑ + 1/2O_2_↑

The initial stage of thermal runaway is simulated as a CO_2_/C_2_H_4_/O_2_ mixed gas with mole fractions of 0.4/0.4/0.2. Separation is performed using the vacuum swing adsorption (*VSA*) process, with adsorption at 1 bar and desorption at 0.1 bar. The operating temperature is 363 K (approximately 90 °C).

To describe the adsorbate-adsorbate interactions, the universal force field was applied to the MOFs [34]. The interactions between adsorbates were described using the Lorentz-Berthelot mixing rules. In the calculations for each structure, the supercell of the MOF was used, ensuring that the simulation box had dimensions at least twice the cutoff radius along each crystallographic direction. Non-bonded interactions were represented by the Lennard-Jones potential with a cutoff radius of 12.8 Å, while Coulombic interactions were computed using the Ewald summation method, with a relative error of 10^6^. The Monte Carlo moves in the GCMC simulation included translation, rotation, addition/deletion, re-insertion, and identity change, all attempted with equal probability. The initialization run was set to a total of 1 × 10^4^ cycles, followed by 3 × 10^4^ cycles for the production run.

### 2.3. Calculation of Chemical Descriptors by RASPA

All simulations assume rigid MOF structures and equilibrium adsorption under idealized dry gas conditions, which allows internally consistent comparison across chemically diverse MOF datasets. Framework flexibility, humidity, electrolyte vapors, and time-dependent response will be addressed during device-level evaluation rather than at the material screening stage. The same assumptions are applied uniformly to all candidate materials, ensuring that the identified trends and structure-property relationships for CO_2_ recognition are robust within the defined operating conditions.

Adsorption-related chemical descriptors were calculated using the RASPA2.0 software [35]. These descriptors include Henry’s law coefficients (*K_H_*) and adsorption enthalpies at infinite dilution (*Q*^0^*_st_*) for CO_2_, C_2_H_4_, and O_2_, which collectively describe adsorption affinity and energetic interactions under low-pressure conditions. Henry’s law coefficient *K_H_* reflects the initial adsorption affinity and governs competitive uptake behavior in the low-pressure regime relevant to early thermal runaway conditions. In contrast, *Q*^0^*_st_* characterizes the intrinsic interaction strength between gas molecules and the framework and provides thermodynamic insight into adsorption selectivity. *K_H_* values were obtained using the Widom test particle insertion method (with a Widom probability set to 1) with 10,000 Monte Carlo cycles, while *Q*^0^*_st_* values were calculated from additional simulations of 20,000 cycles under 367 K [36].

To account for moisture effects that may arise in realistic battery environments, hydrophobicity was introduced as an explicit screening constraint. *K_H_*(H_2_O) was calculated for all 1470 candidate MOFs at 363 K using the same Widom insertion protocol. Hydrophobic materials were identified using a threshold of *K_H_*(H_2_O) < 2.6 × 10^−6^ mol/(kg·Pa), as established in previous studies [23]. Applying this criterion effectively excludes hydrophilic structures and ensures that the selected candidates possess intrinsic resistance to water interference. Supporting Information (SI) and Table S2 provide other relevant chemical descriptors.

### 2.4. Adsorption Performance Metrics

Three evaluation metrics were employed: the adsorption selectivity of CO_2_ over C_2_H_4_ and O_2_ (*S*), the working capacity of CO_2_ (Δ*N*), and the trade-off between working capacity and selectivity (*TSN*) [23]. The specific equations are presented in Table 1, where $x_{i}$ and $y_{i}$ represent component i in the adsorption and gas phase, respectively. $N_{CO_{2}}^{ads}$ and $N_{CO_{2}}^{des}$ are the CO_2_ loading under the adsorption and desorption pressures, respectively.

## 3. Result and Discussion

### 3.1. Distributions of Structural Descriptors

**Figure 1:**
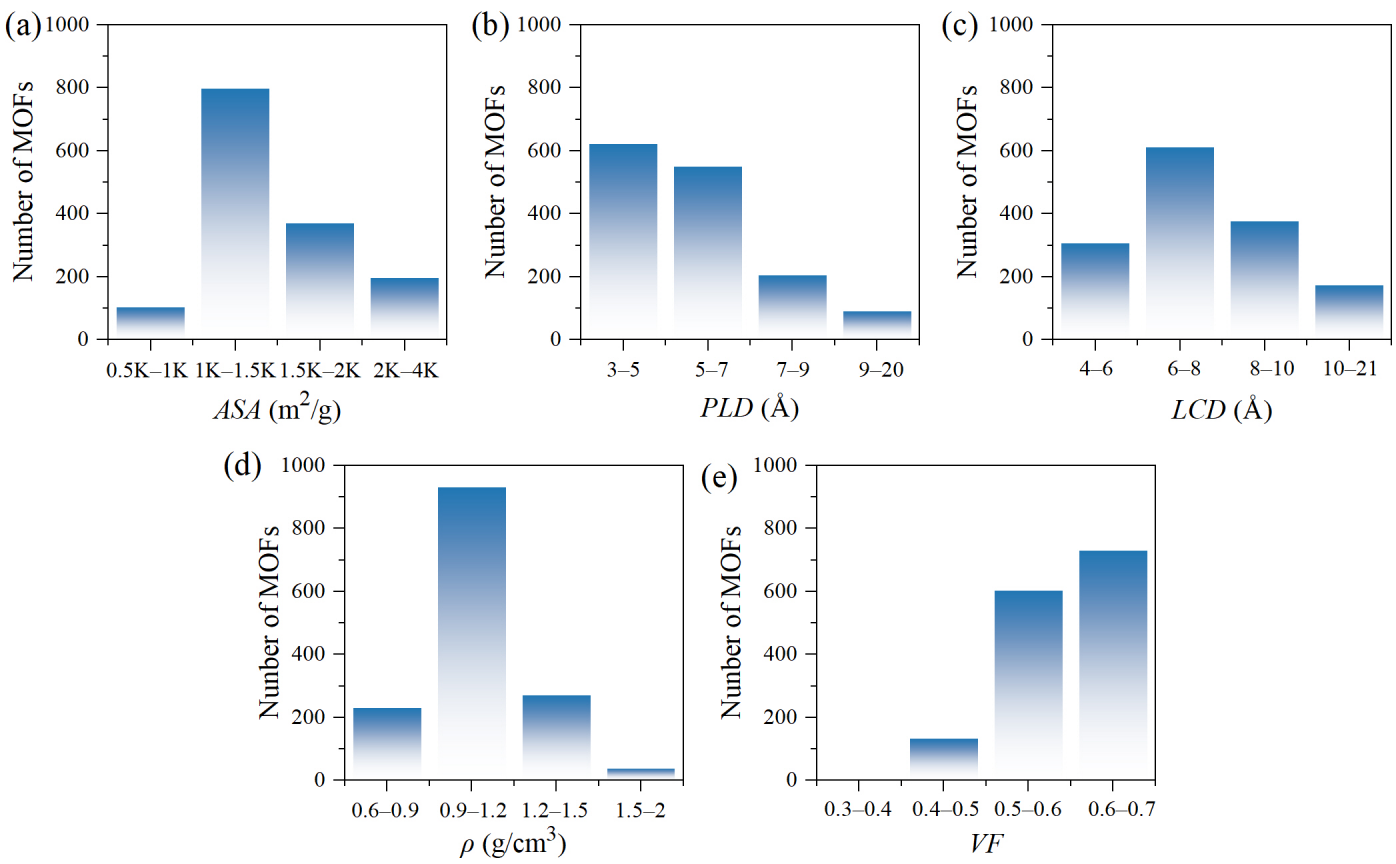
Histograms showing the distribution of key structural descriptors for 1470 MOFs: (**a**) *ASA*, (**b**) *PLD*, (**c**) *LCD*, (**d**) *ρ*, and (**e**) *VF*.

The structural diversity of the 1470 MOFs in the dataset plays a critical role in ensuring robust ML models with strong generalization capability. Figure 1 presents histograms of five key structural descriptors. These distributions cover broad value ranges and align well with the requirements for selective CO_2_ adsorption under low-pressure competitive conditions. *ASA* is a key factor influencing physisorption capacity, with values ranging from around 500 to over 4000 m^2^/g. Most materials cluster between 1000 and 2000 m^2^/g as shown in Figure 1a. This wide distribution enables the model to capture the complex relationship between surface area and performance. This challenges the oversimplified view that higher *ASA* always leads to higher loading [37]. MOFs with *PLD* in the 3–7 Å range are the most abundant (Figure 1b), while those with *LCD* show a broader distribution extending up to approximately 20 Å (Figure 1c). The target gases have kinetic diameters of 3.3 Å for CO_2_, 3.9 Å for C_2_H_4_, and 3.64 Å for O_2_. Most MOFs therefore possess pore dimensions closely matching these molecular sizes, providing the structural basis for molecular sieving and diffusion selectivity. Pores significantly smaller than these dimensions restrict gas entry, while excessively large pores diminish selectivity [38]. The wide *PLD* and *LCD* distributions allow the model to learn structure-performance relationships across various architectures, rather than being limited to a narrow structural range. The highest number of MOFs falls within the *ρ* range of 0.9 to 1.2 g/cm^3^ (Figure 1d) indicating greater framework compactness, which typically correlates inversely with porosity. *VF* remains predominantly high, with most materials between 0.5 and 0.7 (Figure 1e), indicating substantial internal space for gas adsorption. These descriptors collectively capture the structural features governing CO_2_ selectivity in competitive adsorption scenarios.

Key descriptors span a wide range of values, which enables ML models to capture diverse structure-performance relationships across various MOF architectures, rather than being restricted to specific structural types. The pore size distributions are primarily concentrated in the microporous range, matching the dimensions of target gases like CO_2_ and C_2_H_4_. Along with favorable pore volume and surface area distributions, these features highlight the database’s inclusion of numerous materials with strong adsorption potential. This structural diversity not only enhances model generalization but also supports the rational and application-oriented nature of the data selection process.

### 3.2. Descriptor-Dependent Adsorption Trends in Low-Pressure Multicomponent CO_2_ Recognition

Structure-performance relationships reveal non-intuitive trends for low-pressure multicomponent CO_2_ recognition (Figure S1). Working capacity does not show a simple positive correlation with *ASA*, *ρ*, or *VF*. Instead, top performers are found at moderate *ASA* values between 950 and 1750 m^2^/g. They also fall within optimal pore ranges of *PLD* (4–8 Å) and *LCD* (5–9 Å), balancing diffusion access with strong van der Waals interactions (Figure S1). Selectivity favors smaller pores and lower *ASA* to enhance molecular confinement and restrict interfering gases (Figure S1). The composite metric *TSN* integrates both capacity and selectivity, which exhibits the sharpest structural dependencies. High-performing MOFs occupy narrow pore size ranges of 4–7 Å *PLD* and 5.5–9.6 Å *LCD*, combined with moderate *ASA*, high *VF* above 0.6, and low *ρ* below 1.3 g/cm^3^ (Figure S1).

These findings differ markedly from traditional high-pressure single-component adsorption studies which uptake scales directly with surface area. In the targeted low-partial-pressure competitive environment, efficient site utilization and precise pore tuning outweigh any single descriptor. ML uncovers these nuanced structure-performance relationships that conventional heuristic screening would miss. This enables the identification of MOF candidates optimized for selective CO_2_ recognition in early lithium-ion battery thermal runaway warning systems.

**Figure 2:**
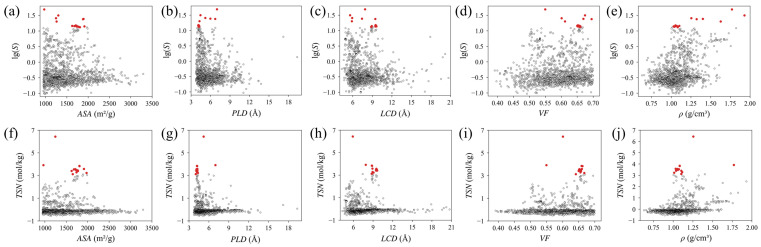
Scatter plots showing the relationship between CO_2_ selectivity (lg(*S*)) and five key structural descriptors: (**a**) *ASA*, (**b**) *PLD*, (**c**) *LCD*, (**d**) *VF*, (**e**) *ρ*. The relationship between *TSN* and five key structural descriptors: (**f**) *ASA*, (**g**) *PLD*, (**h**) *LCD*, (**i**) *VF*, (**j**) *ρ*. The top 15 MOFs are highlighted as red points, and the remaining MOFs are shown in gray points.

Selectivity exhibits distinct structural dependencies favoring confined environments (Figure 2). Although selectivity generally increases as *ASA* decreases, the highest-performing MOFs cluster at moderate values rather than at the minimum extremes (Figure 2a). Excessively open structures reduce confinement effects that enhance CO_2_ binding relative to C_2_H_4_ and O_2_. Furthermore, pore dimensions strongly influence selectivity, with performance decreasing as pore sizes increase (Figure 2b,c). Top-performing MOFs cluster within a moderately small pore range that provides sufficient confinement to strengthen CO_2_ interactions without restricting diffusion. In contrast, *VF* and *ρ* show less pronounced correlations with selectivity (Figure 2d,e), though high performers still favor moderately high *VF* above 0.5 and low *ρ* below 2 g/cm^3^. These trends indicate that superior CO_2_ selectivity under competitive low-pressure conditions emerges from balanced pore confinement that optimizes interaction strength differences among competing gases. Consequently, such selectivity is governed by these discrete local environments rather than the broad maximization of surface area or pore volume.

*TSN* shows clear structural dependencies for selective CO_2_ recognition under thermal runaway conditions. High-performing MOFs concentrate in the moderate *ASA* range of approximately 965 to 1975 m^2^/g (Figure 2f). This range balances CO_2_ working capacity with selectivity against C_2_H_4_ and O_2_. While increasing *ASA* provides more total adsorption sites, it simultaneously compromises selectivity by facilitating indiscriminate gas uptake. Pore size descriptors *PLD* and *LCD* exhibit sharply peaked relationships with *TSN*, revealing a narrow optimal window (Figure 2g,h). High-*TSN* materials cluster almost exclusively within *PLD* ≈ 4–7 Å and *LCD* ≈ 5.5–9.6 Å. Within these specific dimensions, pore confinement effects preferentially strengthen CO_2_ adsorption relative to the slightly larger C_2_H_4_ (3.9 Å) and O_2_ (3.64 Å). This behavior confirms that selectivity originates from enhanced interaction strengths within confined spaces rather than simplistic size exclusion. Conversely, *TSN* rapidly declines when pore dimensions exceed *PLD* > 7 Å or *LCD* > 10 Å, as enlarged channels permit competing gases to adsorb with similar affinities. *TSN* also shows clear preferences for *VF* and *ρ* (Figure 2i,j). High-performing MOFs favor high *VF* above 0.6 and low framework densities below 1.3 g/cm^3^. Such a combination ensures maximum site exposure and mass transfer efficiency without sacrificing gravimetric capacity. Collectively, these trends establish a precise profile for MOF design: moderate surface area, 4–7 Å pore windows, and high-porosity, low-density frameworks. This structural profile holds promise for providing explicit guidance in developing CO_2_ sensors for battery safety applications.

### 3.3. Predictive Performance of the RF Model

This study systematically compared three machine learning algorithms, including Decision Tree (DT), Random Forest (RF), and Extreme Gradient Boosting (XGBoost), to identify a robust predictive model for CO_2_/C_2_H_4_/O_2_ separation performance. The dataset was randomly divided into a training set comprising 80% of the data and an independent testing set comprising the remaining 20%. Bayesian hyperparameter optimization combined with 10-fold cross validation was applied during model training to improve robustness and reduce the risk of overfitting. The predictive performance of the optimized models on the independent testing set is summarized in Table S3. Although XGBoost achieved extremely high fitting accuracy on the training set with *R*^2^ values exceeding 0.99, its performance declined markedly on the testing set, with a testing *R*^2^ of approximately 0.82 for lg(*S*). This behavior indicates limited generalization capability. In contrast, the RF model demonstrated superior generalizability and consistency across all individual metrics. For instance, RF model maintained high and stable testing *R*^2^ values for Δ*N* (0.929), lg(*S*) (0.937), and *TSN* (0.920), consistently outperforming or matching other models in the testing phase. Based on this robust balance between predictive accuracy and generalizability, the RF model was selected for subsequent analysis.

**Figure 3:**
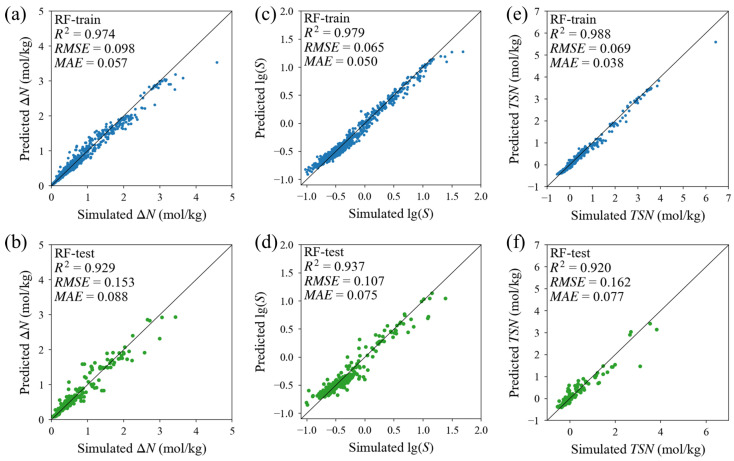
Comparison between predicted and simulated values of (**a**,**b**) Δ*N*, (**c**,**d**) lg(*S*), and (**e**,**f**) *TSN* on the training and testing sets using the RF model.

Detailed evaluation of the RF model (Figure 3) confirms its high predictive efficacy. For working capacity Δ*N*, the narrow gap between training and testing metrics signifies the model’s ability to internalize intrinsic structure-property relationships rather than merely memorizing data (Figure 3a,b). Predictive accuracy is further enhanced for lg(*S*) (*R*^2^ = 0.937), where the logarithmic transformation successfully normalized the distribution to facilitate the precise identification of highly selective MOFs (Figure 3d). Finally, the composite *TSN* metric exhibits strong performance (*R*^2^ = 0.920) with data points tightly clustered around the diagonal (Figure 3e,f). This high accuracy across all derived metrics validates the credibility of the top-ranked candidates identified from the database (Tables S4 and S5).

The strong predictive consistency of the RF model across all three metrics confirms that *TSN* can be reliably used as a screening indicator linking adsorption capacity and selectivity. This level of predictive accuracy provides a data-driven basis for identifying MOFs that simultaneously exhibit high CO_2_ working capacity and strong competitive adsorption behavior.

### 3.4. Interpretation of Structure-Property Relationships Using SHAP Analysis

**Figure 4:**
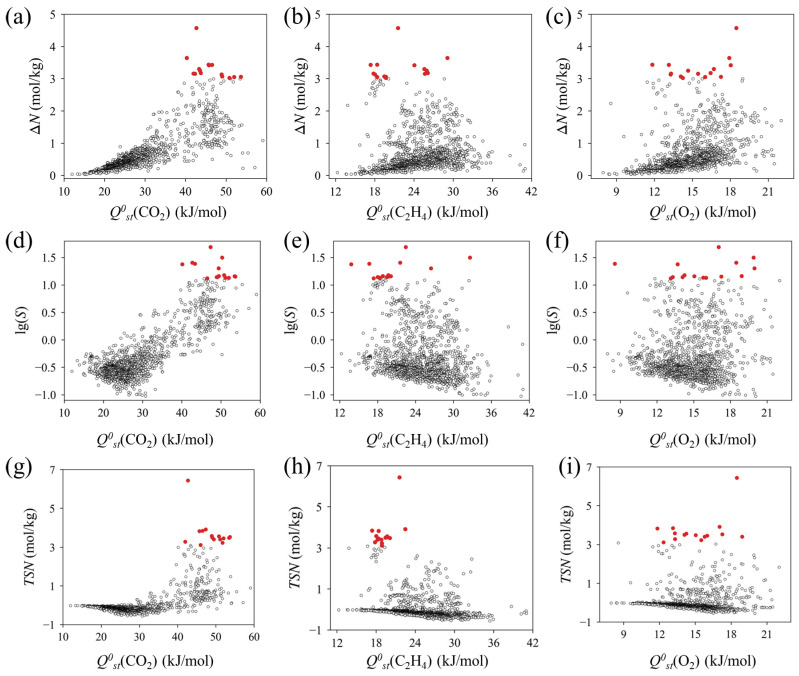
Correlations between performance metrics and infinite-dilution isosteric heats of adsorption *Q*^0^*_st_*. (**a**–**c**) Working capacity (Δ*N*) versus *Q*^0^*_st_* for CO_2_, C_2_H_4_, and O_2_, respectively. (**d**–**f**) lg(*S*) versus *Q*^0^*_st_* for CO_2_, C_2_H_4_, and O_2_, respectively. (**g**–**i**) *TSN* versus *Q*^0^*_st_* for CO_2_, C_2_H_4_, and O_2_, respectively. The top 15 MOFs are highlighted as red points, and the remaining MOFs are shown in gray points.

Prior to the multivariate SHAP analysis, we examined the univariate correlations to establish the fundamental physical bounds governing separation performance. As shown in Figure 4a–c, Δ*N* displays a non-monotonic, volcano-like trend relative to *Q*^0^*_st_*. This behavior aligns with the Sabatier principle, reflecting a critical balance that interactions must be strong enough to drive uptake but moderate enough to facilitate efficient regeneration [39]. For CO_2_, the highest capacities occur within an optimal window (~42.7 kJ/mol) instead of the theoretical maximum (Figure 4a). Similarly, C_2_H_4_ capacity peaks at an intermediate *Q*^0^*_st_* (C_2_H_4_) of 21.6 kJ/mol (Figure 4b). These results suggest that excessive binding strength forms a thermodynamic trap that hinders desorption. In terms of selectivity, the correlation profiles reveal a distinct asymmetry in Figure 4d–f. While high CO_2_ affinity defines the upper bound of selectivity (Figure 4d), the data in Figure 4e shows that C_2_H_4_ binding strength acts as a critical threshold. All highly selective MOFs are strictly confined to a low-enthalpy region (*Q*^0^*_st_*(C_2_H_4_) < 30 kJ/mol), beyond which selectivity suffers a sharp attenuation due to the competitive occupancy of adsorption sites by C_2_H_4_. While O_2_ affinity plays a negligible role (Figure 4f), the performance ceiling depends on a trade-off. Specifically, CO_2_ attraction must be maximized while a rigorous low-affinity boundary is maintained for C_2_H_4_ [40,41]. Synthesizing these factors, the composite *TSN* metric in Figure 4g,h identifies a clear optimal spot centered at *Q*^0^*_st_*(CO_2_) 42.7 kJ/mol and *Q*^0^*_st_*(C_2_H_4_) between 15 and 24 kJ/mol. This threshold-driven behavior indicates that enhanced CO_2_ affinity defines the performance ceiling. However, the minimization of C_2_H_4_ interference acts as the decisive limiting factor for practical viability, while O_2_ adsorption plays only a negligible secondary role (Figure 4i).

**Figure 5:**
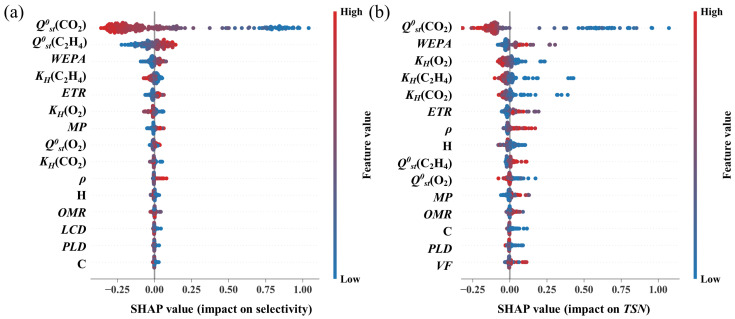
SHAP value distributions for (**a**) selectivity and (**b**) *TSN* based on the RF model.

SHAP analysis further elucidates the relative contribution of each descriptor. As shown in Figure 5a, the feature ranking identifies *Q*^0^*_st_*(CO_2_), *Q*^0^*_st_*(C_2_H_4_), *WEPA*, *K_H_*(C_2_H_4_), and *ETR* as the dominant drivers governing selectivity. Specifically, *Q*^0^*_st_* descriptors play a decisive role in the competitive landscape. High *Q*^0^*_st_*(CO_2_) values correspond to strongly positive SHAP contributions, confirming that robust adsorbate-framework interactions enable CO_2_ to dominate adsorption sites. Conversely, elevated *Q*^0^*_st_*(C_2_H_4_) values shift the SHAP distribution toward the negative regime, indicating that heightened ethylene affinity severely undermines discriminatory power. This mechanism is reinforced by the *K_H_* descriptors: minimizing the baseline affinity for competitors *K_H_*(C_2_H_4_) and *K_H_*(O_2_) is critical for maintaining a CO_2_-selective environment, particularly at low partial pressures.

Electronic descriptors *WEPA* and *ETR* jointly govern the polarity-related characteristics of MOF frameworks at different structural scales and exert a dominant influence on CO_2_ selectivity [42,43]. *WEPA* reflects the overall electronegativity background of the framework, determined by the averaged electron-withdrawing contributions of its constituent atoms. Higher *WEPA* values reflect frameworks enriched with electronegative atoms (O, N, F). These increase global polarity and strengthen electrostatic interactions with the CO_2_ quadrupole. In contrast, *ETR* characterizes the density of electronegative atoms acting as localized polar sites. These create concentrated electric fields that promote directional electrostatic or Lewis acid-base interactions with CO_2_. Owing to the much weaker electrostatic response of C_2_H_4_, these highly polar environments preferentially amplify CO_2_ binding and thus enhance selectivity [44].

Regarding the composite metric *TSN*, *Q*^0^*_st_*(CO_2_) emerges as the primary determinant and exerts a profound positive influence (Figure 5b). Intensifying MOF-CO_2_ interactions simultaneously amplifies both adsorption capacity and selectivity, thereby compounding their effect on the final *TSN* value. Similarly, *WEPA* contributes positively by selectively enhancing the capture of quadrupolar CO_2_. In contrast, *K_H_*(O_2_) and *K_H_*(C_2_H_4_) display strong negative correlations, where minimized affinity for non-target species is essential to preserve *TSN*. A counterintuitive finding is the negative influence of *K_H_*(CO_2_), with optimal *TSN* scores clustering at moderate-to-low values. Extremely high *K_H_*(C_2_H_4_) can indicate ultra-strong sites that attract polarizable C_2_H_4_, creating a competitive binding effect that reduces overall selectivity.

### 3.5. Mechanistic Insights and Structural Feasibility of Top Candidates

**Figure 6:**
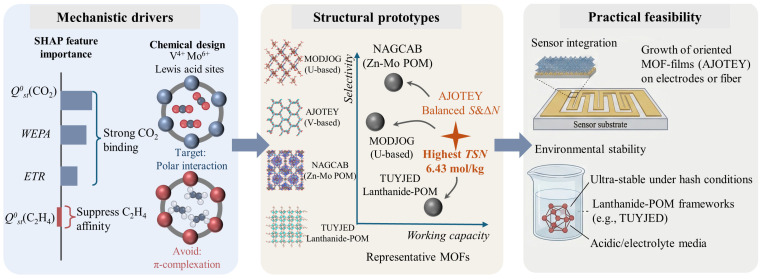
Mechanistic, structural, and practical determinants of high-performance MOF candidates for selective CO_2_ recognition.

Consistent with physical interpretations, SHAP value analysis reveals that optimal MOFs must simultaneously strengthen CO_2_ adsorption while disfavoring C_2_H_4_ uptake. High positive SHAP contributions from *Q*^0^*_st_*(CO_2_), *WEPA*, and *ETR* confirm that a polar framework provides the fundamental basis for strong CO_2_ binding (Figure 6). In contrast, the strongly negative SHAP values of *Q*^0^*_st_*(C_2_H_4_) indicate that suppressing the affinity for competing C_2_H_4_ is equally critical. Chemically, this selective profile is achieved by a specific design strategy: the absence of strong π-complexation sites. The top candidates deliberately avoid low-valent transition metals (e.g., Cu^+^, Ag^+^) and high-density aromatic linkers that could enable π-π stacking with ethylene [40,45,46]. Instead, they predominantly utilize high-valent metal centers (e.g., V^4+^, U^6+^, Mo^5+/6+^, W^6+^) and lanthanides (e.g., Sm^3+^, Nd^3+^). As hard Lewis acid centers within inorganic or POM clusters, these metals optimize CO_2_ quadrupole interactions while suppressing affinity for non-polar O_2_ and weakly polar C_2_H_4_ [41,47].

This balance is perfectly exemplified by AJOTEY, the top-performing candidate (*TSN* = 6.43 mol/kg) [48]. Structurally, AJOTEY is a V^4+^-based framework constructed from vanadium-oxo clusters and phosphito linkers. The high density of polar V=O and V-OH sites serves as directional CO_2_ binding centers [49]. Geometrically, it aligns with the identified optimal profile, featuring a *PLD* of 5.20 Å, *LCD* of 5.91 Å, moderate *ASA* of 1245.70 m^2^/g, and high *VF* (0.60). Statistical validation across the top 15 candidates reinforces the importance of this intermediate pore window (average *PLD* ~4.5 Å), which successfully balances molecular sieving effects with the accessibility required for robust gas transport.

The trade-off between selectivity and capacity is evident when comparing specific candidates. NAGCAB (a Zn-Mo POM-based MOF) achieves the highest selectivity of 49.10 but ranks only second in *TSN* due to its modest working capacity (Tables S4 and S5). Its relatively small pores (*PLD* 6.99 Å) strongly enhance selectivity through confinement but limit total uptake. Conversely, AJOTEY balances both metrics by sacrificing some selectivity to achieve nearly double the working capacity (Figure 6). Similarly, NAQRAA and NAQREE (Sm/Eu-based POM-MOFs) adopt smaller *PLD* values (~4.2 Å) with larger *LCD* (~8.8 Å), creating elongated pore geometries that sustain high selectivity while preserving essential void space [50,51,52]. In contrast, MODJOG (a U-based framework) maximizes selectivity at the expense of capacity, resulting in a lower *TSN*. These comparisons demonstrate the model’s efficacy in prioritizing materials that resolve inherent adsorption trade-off.

Finally, regarding practical feasibility, all 15 top candidates are experimentally synthesizable, as evidenced by the CSD. Film formation is particularly promising for sensor integration (Figure 6). Many of these structures, including AJOTEY and POM-based families (like NAGCAB and NAQRAA), offer unique electrostatic properties suitable for deposition on sensor electrodes [49,53]. In terms of stability, the Lanthanide-POM based candidates such as TUYJED (Nd/W-based) and the LEYREO series (Pr/Mo/V-based) provide a robust alternative [52,53]. Rigid polyoxometalate backbones ensure structural integrity in acidic and oxidative environments, offering superior resistance to humidity and electrolyte leakage. Thus, the screened library offers a versatile toolkit: V-based AJOTEY as the high-performance standard, and Lanthanide/POM-MOFs as ultra-stable backups for harsh operating conditions.

## 4. Conclusions

In summary, this research demonstrates an efficient methodology for screening MOFs for early CO_2_ recognition during lithium-ion battery thermal runaway. By integrating GCMC simulations with an RF model, we successfully identified optimal materials capable of distinguishing CO_2_ from C_2_H_4_ and O_2_ under competitive conditions. SHAP analysis reveals that performance is governed by a delicate balance of adsorption energetics and pore accessibility. Specifically, CO_2_ selectivity originates from interaction strength differences driven by hard Lewis acid centers and polar inorganic clusters rather than simple size exclusion. Our findings indicate that high-performing MOFs do not possess extreme structural traits but instead fall within a narrow, balanced design window: moderate *ASA* (965–1975 m^2^/g), constrained pore openings (*PLD* ≈ 4–7 Å), high *VF* (>0.6), and low framework *ρ* (<1.3 g/cm^3^). Among the candidates, AJOTEY stands out by resolving the inherent capacity-selectivity trade-off, achieving a superior *TSN* value (6.43 mol/kg). Furthermore, the identification of Lanthanide-POM based frameworks provide versatile ultra-stable alternatives suitable for the harsh chemical environments of battery packs. This computational workflow significantly accelerates the discovery of sensing materials and establishes a reliable foundation for developing highly selective CO_2_ sensors to enhance battery safety systems.

## Figures and Tables

**Table 1:** Metrics used to evaluate the MOF adsorbents.

Performance Metrics	Formula
Adsorption selectivity	$S=(x_{CO_{2}}/x_{C_{2}H_{4}+O_{2}})/(y_{CO_{2}}/y_{C_{2}H_{4}+O_{2}})$
Working capacity	$\Delta N=N_{CO_{2}}^{ads}-N_{CO_{2}}^{des}$
A trade-off between the above two metrics	$TSN=\Delta N\times \log_{10}(S)$

## Data Availability

The raw data supporting the conclusions of this article will be made available by the authors on request.

## Supplementary Materials

The following supporting information can be downloaded at: https://www.mdpi.com/article/10.3390/nano16040245/s1, Table S1: Descriptors characterizing structural and chemical properties of MOFs; Table S2: Calculation of other chemical descriptors; Table S3: The prediction scores of ML models for individual metrics; Table S4: Basic properties and separation performances of the fifteen top-performing MOFs sorted by selectivity; Table S5: Basic properties and separation performances of the fifteen top-performing MOFs sorted by *TSN*; Figure S1: Scatter plots showing the relationship between working capacity (Δ*N*) and five key structural descriptors (*ASA*, *PLD*, *LCD*, *ρ*, *VF*). The top 15 MOFs are highlighted as red points, and the remaining MOFs are shown in gray points.
