# Supplementary material for: Leveraging Machine Learning for Screening Metal-Organic Frameworks with Selective CO2 Recognition for Early Thermal Runaway in Lithium-Ion Batteries
Source: Nanomaterials (Basel). 2026 Feb 13;16(4):245. doi: 10.3390/nano16040245 (PMC12942749; doi:10.3390/nano16040245)
Supplement: Supplementary file 1 [file nanomaterials-16-00245-s001.zip › nanomaterials-4130234-supplementary.pdf]

Supporting information

Leveraging Machine Learning for Screening Metal-Organic Frameworks with Selective CO_2 Recognition for Early Thermal Runaway in Lithium-Ion Batteries

Xian Wei[1]*, Xin Li[2], Xiong Wang[3], Xiaoyan Liu[2]*, Chen Zhu[2]*

1 College of Electronic and Optical Engineering & College of Flexible Electronics (Future Technology), Nanjing University of Posts and Telecommunications, Nanjing 210023, China

2 College of Integrated Circuit Science and Engineering, Nanjing University of Posts and Telecommunications, Nanjing 210023, China

3 ISCTE Business School, Business Research Unit, University Institute of Lisbon, Lisbon 1649–026, Portugal;

* Correspondence: wxian@njupt.edu.cn (X.W.); xiaoyanliu@njupt.edu.cn (X.L.); zhuchen@njupt.edu.cn (C.Z.)

Preliminary Screening of CoRE-MOF 2019 Database Based on Key Structural Parameters

Prior to performing CO_2 adsorption simulations on MOF materials, it is essential to conduct a preliminary screening of the CoRE-MOF 2019 database based on key structural parameters. When the *PLD* of a MOF is smaller than 3.3 Å (the kinetic diameter of CO_2), CO_2 molecules experience significant difficulty in entering the pore interior, leading to a dramatic reduction in adsorption capacity. The *ASA* represents another critical factor influencing adsorption capacity. A higher *ASA* generally favors enhanced CO_2 uptake, and in most cases, a moderate *ASA* in the range of 1000-2500 m^2/g is sufficient to achieve excellent adsorption performance. Furthermore, the *VF* shows a significant positive correlation with the CO_2 adsorption capacity of MOFs, with materials possessing higher *VF* values typically exhibiting superior adsorption potential. Based on the above findings, the present study establishes the following screening criteria: $PLD \geq 3.3$ Å, 950 $m^2/g < ASA < 5000$ m^2/g, and $0.1 < VF < 0.7$. Applying these criteria, 1470 MOF structures with potential for CO_2 adsorption were successfully identified from the CoRE-MOF 2019 database, thereby providing a solid foundation for subsequent molecular simulations and performance investigations.

Table S1 Descriptors characterizing structural and chemical properties of MOFs

Category	Descriptor	Unit
Structural Descriptors	Largest Cavity Diameter (*LCD*)	Å
	Pore Limiting Diameter(*PLD*)	Å
	Accessible Surface Area (*ASA*)	m^2/g
	Void Fraction (*VF*)	—
	Density (ρ)	g/cm^3
Chemical Descriptors	Henry's Coefficient(K_H)	mol/(kg·Pa)
	Enthalpy of adsorption at infinite dilution(Q_{st}^{0})	kJ/mol
	Number of H Atoms per Unit Cell (*N_H*)	—
	Number of C Atoms per unit cell (*N_C*)	—
	Number of N Atoms per Unit Cell (*N_N*)	—
	Number of F Atoms per Unit Cell (*N_F*)	—
	Number of Cl Atoms per Unit Cell (*N_Cl)*	—
	Number of Br Atoms per Unit Cell (*N_Br*)	—
	Number of V Atoms per Unit Cell (*N_V*)	—
	Number of Cu Atoms per Unit Cell (*N_Cu*)	—
	Number of Zn Atoms per Unit Cell (*N_Zn*)	—
	Number of Zr Atoms per Unit Cell (*N Zr*)	—
	Total Degree of Unsaturation (*TDU*)	—
	Metallic Percentage (*MP*)	—
	Oxygen-to-Metal Ratio (*OMR*)	—
	Electronegative-to-Total Ratio (*ETR*)	—
	Weighted Electronegativity per Atom (*WEPA*)	—
	Nitrogen-to-Oxygen Ratio (*NOR*)	—

Calculation of Other Chemical Descriptors

The calculations are performed using Python scripts, and the formulas are as follows:

Total Degree of Unsaturation (*TDU*):

$$TDU = \frac{2N_{Carbon}+2-N_{Hydrogen}}{2} \tag{S1}$$

Metallic Percentage (*MP*):

$$MP(\%) = \frac{N_{Metal\ Atoms}}{N_{Carbon}} \times 100 \tag{S2}$$

Oxygen to Metal Ratio (*OMR*):

$$OMR = \frac{2N_{Oxygen}}{N_{Metal\ Atoms}} \tag{S3}$$

Electronegative-to-Total Ratio (*ETR*):

$$ETR = \frac{N_{Eletronegative\ Atoms}}{Total\ N_{Atoms}} \tag{S4}$$

Weighted Electronegativity per Atom (*WEPA*):

$$WEPA = \frac{Weighted\ Electronegativity\ Atoms}{Total\ N_{Atoms}} \tag{S5}$$

Nitrogen-to-Oxygen Ratio (*NOR*):

$$NOR = \frac{N_{Nitrogen}}{N_{Oxygen}} \tag{S6}$$

Table S2 Calculation of other chemical descriptors.

Descriptor	Calculation Note
Number of H Atoms per Unit Cell (*N_H*)	number of hydrogen atoms per unit cell
Number of C Atoms per unit cell (*N_C*)	number of carbon atoms per unit cell
Number of N Atoms per Unit Cell (*N_N*)	number of nitrogen atoms per unit cell
Number of F Atoms per Unit Cell (*N_F*)	number of oxygen atoms per unit cell
Number of Cl Atoms per Unit Cell (*N_Cl)*	number of fluorine atoms per unit cell
Number of Br Atoms per Unit Cell (*N_Br*)	number of chlorine atoms per unit cell
Number of V Atoms per Unit Cell (*N_V*)	number of bromine atoms per unit cell
Number of Cu Atoms per Unit Cell (*N_Cu*)	number of vanadium atoms per unit cell
Number of Zn Atoms per Unit Cell (*N_Zn*)	number of copper atoms per unit cell
Number of Zr Atoms per Unit Cell (*N Zr*)	number of zinc atoms per unit cell
Total Degree of Unsaturation (*TDU*)[a]	$TDU=\frac{2N_{Carbon}+2-N_{Hydrogen}}{2}$
Metallic Percentage (*MP*)	$MP(\%)=\frac{N_{Metal\ Atoms}}{N_{Carbon}}\times 100$
Oxygen-to-Metal Ratio (*OMR*)	$OMR=\frac{2N_{Oxygen}}{N_{Metal\ Atoms}}$
Electronegative-to-Total Ratio (*ETR*)	$ETR=\frac{N_{Eletronegative\ Atoms}}{Total\ N_{Atoms}}$
Weighted Electronegativity per Atom (*WEPA*)[b]	$WEPA=\frac{Weighted\ Electronegativity\ Atoms}{Total\ N_{Atoms}}$
Nitrogen-to-Oxygen Ratio (*NOR*)	$NOR=\frac{N_{Nitrogen}}{N_{Oxygen}}$

[a]In the *TDU* formula, for other elements: oxygen is ignored; halogens (F, Cl, Br, I) are treated as hydrogen, and nitrogen is counted as half of carbon.

[b]In the *WEPA* formulaElectronegative atoms: O, N, F, Cl, and Br weighted by electronegativity.

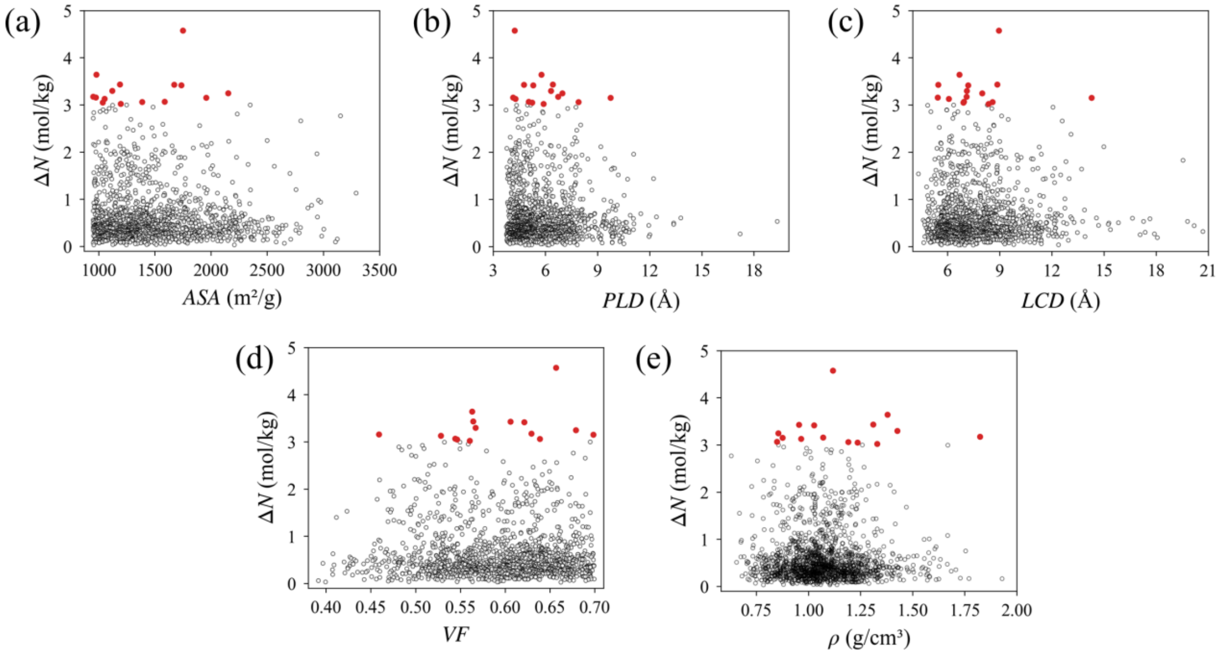

Figure S1 Scatter plots showing the relationship between working capacity (ΔN) and five key structural descriptors (ASA, PLD, LCD, ρ, VF). The top 15 MOFs are highlighted as red points, and the remaining MOFs are shown in gray points.

Comparison of ML Algorithms

Ten-fold cross-validation was employed to prevent overfitting and assess the generalizability of the predictive model. The MOF structures were randomly split into training (80%) and testing sets (20%). A total of five structural descriptors and eighteen chemical descriptors were calculated for the MOFs, with Henry coefficients and adsorption enthalpy under infinite dilution conditions for all three gases included. Hyperparameters for the three ML algorithms were optimized using Bayesian optimization. The performance of the algorithms was evaluated using the coefficient of determination (R^2), mean absolute error (*MAE*), and root mean squared error (*RMSE*). The formulas are as follows:

$$R^2 = 1 - \frac{\sum_{i=1}^{n}(x_i - y_i)^2}{\sum_{i=1}^{n}(y_i - \bar{y})^2} \tag{S7}$$

$$MAE = \frac{1}{n}\sum_{i=1}^{n} |x_i - y_i| \tag{S8}$$

$$RMSE = \frac{1}{n}\sum_{i=1}^{n} (x_i - y_i)^2 \tag{S9}$$

Table S3 The prediction scores of ML models for individual metrics.

Metrics	Prediction metrics	XGBoost		RF		DT	
		Training	Testing	Training	Testing	Training	Testing
ΔN (mol/kg)	R^2	0.993	0.932	0.974	0.929	0.908	0.847
	RMSE	0.051	0.149	0.098	0.153	0.184	0.223
	MAE	0.033	0.087	0.057	0.088	0.113	0.14
lg(S)	R^2	0.999	0.822	0.979	0.937	0.963	0.913
	RMSE	0.101	0.979	0.065	0.107	0.086	0.126
	MAE	0.061	0.26	0.050	0.075	0.061	0.091
TSN (mol/kg)	R^2	0.996	0.94	0.988	0.920	0.851	0.796
	RMSE	0.042	0.14	0.069	0.162	0.241	0.258
	MAE	0.024	0.066	0.038	0.077	0.089	0.11

Table S4 Basic properties and separation performances of the fifteen top-performing MOFs sorted by selectivity.

NO.	CSD code	*PLD* (Å)	*LCD* (Å)	*VF*	ρ (g/cm^3)	*ASA* (m^2/g)	*S*	ΔN (mol/kg)	*TSN* (mol/kg)
1	NAGCAB	6.99	7.90	0.55	1.77	969.14	49.10	2.32	3.92
2	MODJOG	4.45	5.59	0.67	1.93	1299.96	31.54	1.64	2.46
3	AJOTEY	5.20	5.91	0.60	1.26	1245.70	25.52	4.57	6.43
4	ja905009e_si_002	5.97	7.43	0.67	1.40	1892.23	24.28	2.23	3.09
5	MAWFIC	6.73	9.46	0.70	1.32	1879.82	23.80	2.17	2.99
6	SOKWUM	4.30	5.86	0.61	1.63	1260.34	20.08	1.78	2.32
7	LEYREO	4.15	9.51	0.65	1.05	1710.61	15.08	3.02	3.56
8	LIBQIY	4.10	9.48	0.65	1.10	1624.47	14.52	2.93	3.40
9	FORQIN	4.21	9.58	0.66	1.06	1730.40	14.49	3.00	3.48
10	LEYQUD	4.12	9.49	0.65	1.07	1677.38	14.27	3.06	3.53
11	TUYJED	4.26	8.92	0.66	1.03	1912.17	13.96	3.13	3.58
12	LEYRAK	4.16	9.52	0.65	1.05	1729.19	13.88	3.07	3.50
13	FORQEJ	4.27	9.63	0.66	1.04	1774.36	13.66	3.00	3.40
14	FORPOS	4.25	9.45	0.66	1.04	1765.59	13.56	3.05	3.46
15	NAQRAA	4.19	8.82	0.66	1.08	1805.87	13.24	3.43	3.84

Table S5 Basic properties and separation performances of the fifteen top-performing MOFs sorted by *TSN*.

NO.	CSD code	*PLD* (Å)	*LCD* (Å)	*VF*	ρ (g/cm^3)	*ASA* (m^2/g)	*S*	ΔN (mol/kg)	*TSN* (mol/kg)
1	AJOTEY	5.20	5.91	0.60	1.26	1245.70	25.52	4.57	6.43
2	NAGCAB	6.99	7.90	0.55	1.77	969.14	49.10	2.32	3.92
3	NAQRAA	4.19	8.82	0.66	1.08	1805.87	13.24	3.43	3.84
4	NAQREE	4.22	8.86	0.66	1.08	1810.15	13.03	3.43	3.83
5	TUYJED	4.26	8.92	0.66	1.03	1912.17	13.96	3.13	3.58
6	LEYREO	4.15	9.51	0.65	1.05	1710.61	15.08	3.02	3.56
7	LEYQUD	4.12	9.49	0.65	1.07	1677.38	14.27	3.06	3.53
8	LEYRAK	4.16	9.52	0.65	1.05	1729.19	13.88	3.07	3.50
9	FORQIN	4.21	9.58	0.66	1.06	1730.40	14.49	3.00	3.48
10	FORPOS	4.25	9.45	0.66	1.04	1765.59	13.56	3.05	3.46
11	FORQEJ	4.27	9.63	0.66	1.04	1774.36	13.66	3.00	3.40
12	LIBQIY	4.10	9.48	0.65	1.10	1624.47	14.52	2.93	3.40
13	ZIMLIS	4.21	8.92	0.66	1.11	1738.34	10.94	3.16	3.28
14	NAQQON	4.33	9.08	0.67	1.01	1974.88	12.97	2.90	3.23
15	VEHRIL	4.02	8.80	0.64	1.10	1652.37	11.37	2.95	3.12
